# Neddylation regulates excitatory synaptic transmission and plasticity

**DOI:** 10.1038/s41598-019-54182-2

**Published:** 2019-11-29

**Authors:** Marisa M. Brockmann, Michael Döngi, Ulf Einsfelder, Nils Körber, Damian Refojo, Valentin Stein

**Affiliations:** 1 Institut für Physiologie II, Universität Bonn, Bonn, Germany; 2 Molecular Neurobiology, Max Planck Institute of Psychiatry, Munich, Germany; 3 Laboratorio de Neurobiología Molecular, Instituto de Investigación en Biomedicina de Buenos Aires (IBioBA) - CONICET - Partner Institute of the Max Planck Society, Buenos Aires, Argentina; 4 Present Address: Institut für Neurophysiologie, Charité-Universitätsmedizin, Berlin, Germany

**Keywords:** Molecular neuroscience, Synaptic transmission

## Abstract

Post-translational modifications, like phosphorylation, ubiquitylation, and sumoylation, have been shown to impact on synaptic neurotransmission by modifying pre- and postsynaptic proteins and therefore alter protein stability, localization, or protein-protein interactions. Previous studies showed that post-translational modifications are essential during the induction of synaptic plasticity, defined by a major reorganization of synaptic proteins. We demonstrated before that neddylation, a post-translational modification that covalently binds Nedd8 to lysine-residues, strongly affects neuronal maturation and spine stability. We now analysed the consequences of inhibiting neddylation on excitatory synaptic transmission and plasticity, which will help to narrow down possible targets, to make educated guesses, and test specific candidates. Here, we show that acute inhibition of neddylation impacts on synaptic neurotransmission before morphological changes occur. Our data indicate that pre- and postsynaptic proteins are neddylated since the inhibition of neddylation impacts on presynaptic release probability and postsynaptic receptor stabilization. In addition, blocking neddylation during the induction of long-term potentiation and long-term inhibition abolished both forms of synaptic plasticity. Therefore, this study shows the importance of identifying synaptic targets of the neddylation pathway to understand the regulation of synaptic transmission and plasticity.

## Introduction

Neuronal communication requires presynaptic neurotransmitter release and subsequent postsynaptic receptor activation. To modify synaptic strength, pre- and postsynaptic proteins regulating synaptic transmission are fine-tuned by numerous post-translational modifications^1,2^. Besides the commonly known modifications such as phosphorylation, glycosylation, ubiquitylation, and sumoylation, the conjugation of Nedd8 has been described. We showed earlier that neddylation of non-cullin proteins in neurons is essential for synaptic function^3^. Neddylation describes the process of attaching the ubiquitin-like protein Nedd8 covalently to a specific substrate^4^. Similar to other ubiquitin-like proteins, Nedd8 is covalently bound to lysine residues by an enzymatic cascade consisting of the heterodimeric E1-activating enzyme NAE1 (Nedd8 activating enzyme), the conjugating enzyme Ubc12 and yet to be identified E3-ligases^3–6^. The best-documented function of Nedd8 is to target cullin scaffold proteins, thereby increasing the activity of cullin-RING E3 ubiquitin-ligase complexes (CRLs), which are mainly involved in the control of cell cycle and cellular proliferation^7,8^. Recent reports indicate that neddylation also influences the enzymatic activity, transcriptional function, protein stability, and partner interaction of several non-cullin substrates, suggesting additional functions of Nedd8 conjugation beyond CRLs^3,4,9,10^.

Although Nedd8 was discovered in neurons, only very few neddylated neuronal proteins have been described^11^. Besides Parkin^12^ we discovered that the synaptic protein PSD-95 is neddylated^3^. The discovery of additional targets has been difficult. Several aspects complicate the identification of neddylated proteins, mostly the relative abundance of a neddylated protein is fairly low as target proteins are constantly neddylated and de-neddylated^4^. Unfortunately, a de-neddylase inhibitor has not been discovered. Therefore, analysing the consequences of inhibiting neddylation helps to narrow down possible targets, to make educated guesses, and test specific candidates.

We reported earlier that long-lasting neddylation inhibition in neuronal cultures and genetic mouse models strongly impairs spine development and morphology^3^. We now used acute hippocampal slices and acutely inhibited neddylation for up to 120 min with the specific NAE1 inhibitor MLN-4924^10^. This allowed us to studying functional changes independent of morphological changes, as our initial data indicated that several pre- and postsynaptic proteins are neddylated^3^.

We could show that blocking neddylation in acute brain slices for 120 min does not impact on neuronal excitability or spine morphology. Thus, acute brain slices are ideally suited to investigating the effects of inhibiting neddylation on neurotransmitter release, postsynaptic function, and neuronal plasticity. We show that neddylation is involved in the localization of AMPA and NMDA receptors at the postsynapse. In addition, neddylation regulates presynaptic neurotransmitter release by changing vesicular release probability. Interestingly, blocking de-novo neddylation just during the induction time of LTP and LTD blocks both paradigms for synaptic plasticity. Thus, we could show that protein neddylation is required for synaptic integrity and de-novo neddylation is necessary to induce synaptic plasticity.

## Results

It was previously reported that blocking neddylation of proteins by the NAE1-specific inhibitor MLN-4924^10^ or by genetic ablation of NAE1 specifically in forebrain excitatory neurons decreases spine size and density thus reducing synaptic transmission. We now wanted to study, whether neddylation inhibition impacts on synaptic transmission before morphological changes occur. In addition, we wanted to test whether neddylation of synaptic proteins is involved in the induction of synaptic plasticity by blocking de-novo neddylation during induction time; addressing the question whether neddylation is not only important for neuronal and spine morphology but also for the regulation of synaptic strength.

To address these questions, we used MLN-4924 to inhibit neddylation. The original study describing MLN-4924^10^ shows an IC_50_ for blocking NAE of about 5 nM in a purified enzyme assay. To inhibit neddylation effectively in acute brain slice, we increased the concentration to 1 µM. In our hands, 1 µM MLN-4924 had no side-effect on Ubiquitin-activating enzymes (Supplemental Fig. S1).

### Acute neddylation inhibition does not alter neuronal morphology or excitability in hippocampal brain slices

**Figure 1:**
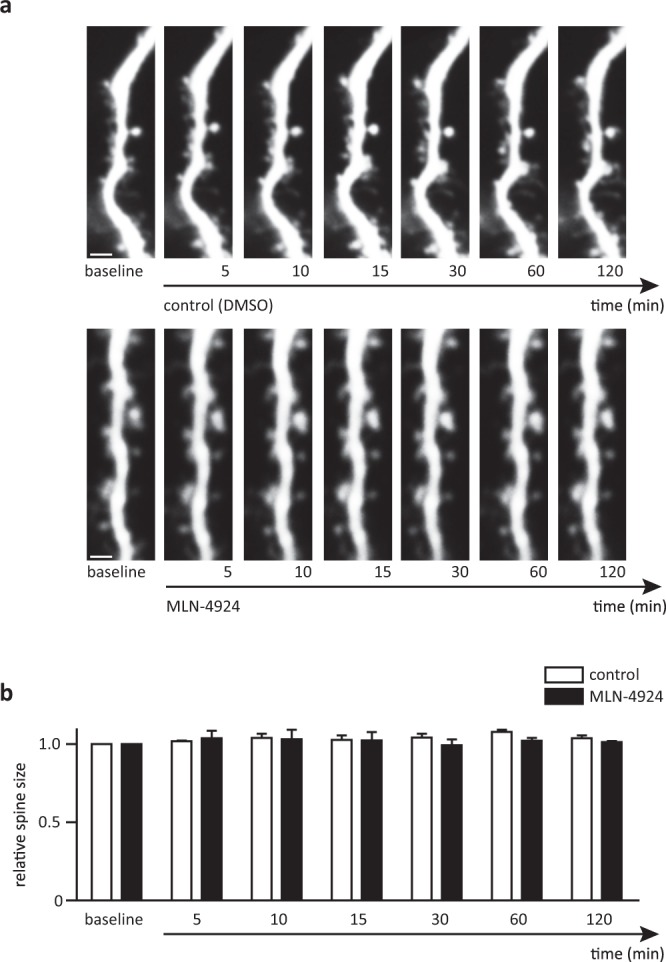
Spine size does not change within 120 min of MLN-4924 treatment. (**a**) Maximum projections of a stretch of dendrite in acute hippocampal slices from Thy-1 GFP mice under control conditions (top) and treated with MLN-4924 (bottom), scale bar 5 µm. (**b**) Relative spine size normalized to the individual spine size before and during MLN-4924 treatment.

In this study, we used acute hippocampal brain slices to investigate the effect of neddylation inhibition on neuronal function. It was essential to exclude changes in spine morphology and neuronal excitability that could influence synaptic transmission. Previous experiments showed that neddylation inhibition in neuronal cultures strongly impacts on spine morphology after 120 min^3^. Therefore, we assessed spine morphology in acute brain slices incubated for 120 min in 1 μM MLN-4924 using two-photon imaging. Acute brain slices were obtained from Thy-1-GFP mice that sparsely express green fluorescent protein (GFP) in hippocampal pyramidal neurons^13^. GFP intensity of spine heads, as a measure for spine size, was monitored over a time course of 120 min after adding MLN-4924. Over the course of 120 minutes, we did not detect any changes in spine size neither in control nor MLN-4924 treated hippocampal slices (Fig. 1a,b, application (Ctrl. Spines n = 115, from 8 slices from 3 animals; MLN Spines n = 135, from 9 slices from 3 animals, values 5 min: Ctrl. 102 ± 4%, MLN 104 ± 5%, 10 min: Ctrl. 104 ± 3%, MLN 103 ± 6%, 30 min: Ctrl. 103 ± 3%, MLN 99 ± 4%, 60 min: Ctrl. 108 ± 2%, MLN 102 ± 2, 120 min: Ctrl. 104 ± 2%, MLN 101 ± 1%, p > 0.05, ANOVA). This finding clearly showed, that spine morphology in acute brain slices is less vulnerable to neddylation inhibition than in neuronal cultures. To avoid morphological effects, we performed all following experiments investigating neuronal transmission within 120 min of MLN-4924 treatment.

**Figure 2:**
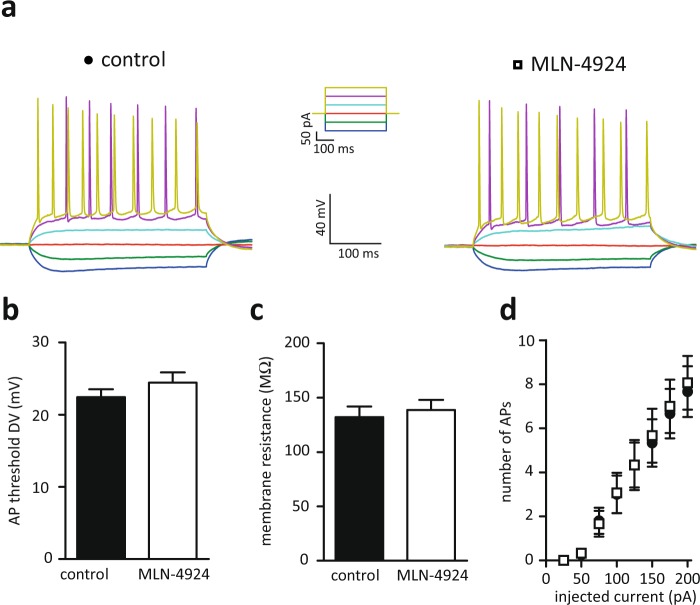
Intrinsic membrane properties are unaltered after MLN-4924 treatment. (**a**) Sample traces of current-clamp recordings; left control (DMSO treated), right after 60 min MLN-4924 treatment. (**b**) the AP threshold is not affected by MLN-4924. (**c**) membrane resistance is not altered by MLN-4924. (**d**) 1 μM MLN-4924 treatment for 60 min does not affect the number of action potentials generated by increasing current injections.

Next, we tested the resting membrane potential and neuronal excitability, parameters that would affect synaptic transmission, of pyramidal hippocampal neurons after 60 min of MLN-4924 treatment. Neddylation inhibition neither affected input resistance nor the action potential threshold. Consistently, the number of action potentials elicited by a super threshold current injection was not changed (Fig. 2b–d, Ctrl. n = 17 cells, MLN n = 15 cells, membrane resistance Ctrl. 139 ± 10 MΩ, MLN 144 ± 14 MΩ, p = 0.754, t-test, AP-threshold Ctrl. 22.5 ± 1 ΔmV, MLN 24.4 ± 2 ΔmV, p = 0.429, t-test). These data indicate that the general health and intrinsic electrophysiological properties of pyramidal hippocampal neurons were not affected by blocking neddylation for 60 minutes.

### Neddylation is required for AMPA and NMDA mediated neurotransmission

**Figure 3:**
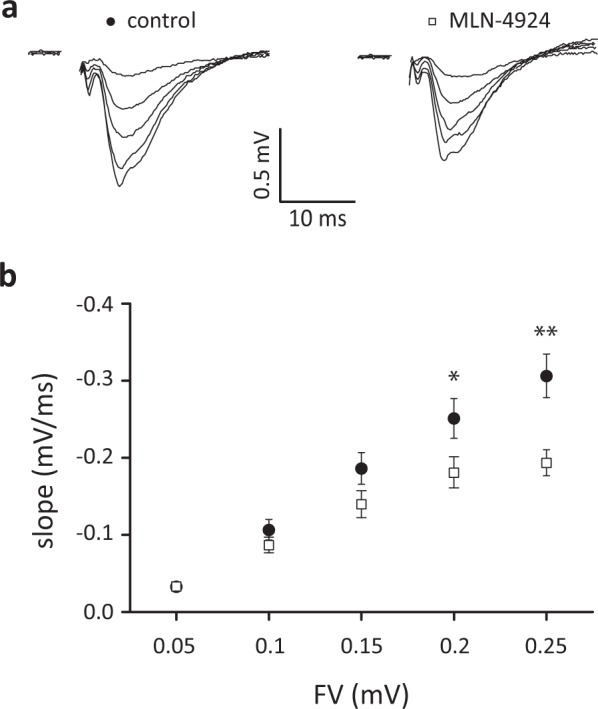
Basal synaptic transmission is reduced in MLN-4924 treated hippocampal brain slices. Input-output curves recorded at the CA3-CA1 synapse show reduced fEPSP responses after 60 min of 1 μM MLN-4924 treatment. (**a**) Representative traces of fEPSCs from control (left) or MLN-4924 (right) treated hippocampal slices using different stimulus intensities. Please note, stimulus artefacts have been omitted for clarity. (**b**) Input-output curve shows fEPSP-slope as a function of stimulus intensity in CA1. fEPSP slopes are significantly lower in MLN-4924 treated slices (Control closed circles, MLN-4924 treated open squares).

To test whether blocking neddylation affects synaptic transmission at CA3-CA1 synapses, we recorded input-output curves in acute hippocampal brain slices treated with DMSO or 1 μM MLN-4924 for at least 30 minutes. We compared the size of the presynaptic fiber volley (input) with the slope of the field EPSP (output) in stratum radiatum. Neddylation inhibition reduced synaptic transmission by ~30% at fiber volleys greater than 0.15 mV (Fig. 3a,b, Ctrl. 0.033 ± 0.006 mV/s, MLN 0.033 ± 0.004 mV/s, p > 0.05, Ctrl. 0.107 ± 0.013 mV/s, MLN 0.087 ± 0.010 mV/s, p > 0.05, Ctrl. 0.186 ± 0.021 mV/s, MLN 0.140 ± 0.017 mV/s, p > 0.05, Ctrl. 0.251 ± 0.026 mV/s, MLN 0.181 ± 0.020 mV/s, p < 0.05, Ctrl. 0.306 ± 0.028 mV/s, MLN 0.193 ± 0.017 mV/s, p < 0.01, 2way ANOVA, Ctrl. n = 9 from 3 animals. MLN n = 10 from 4 animals), indicating that the synaptic transmission is acutely regulated by protein neddylation.

**Figure 4:**
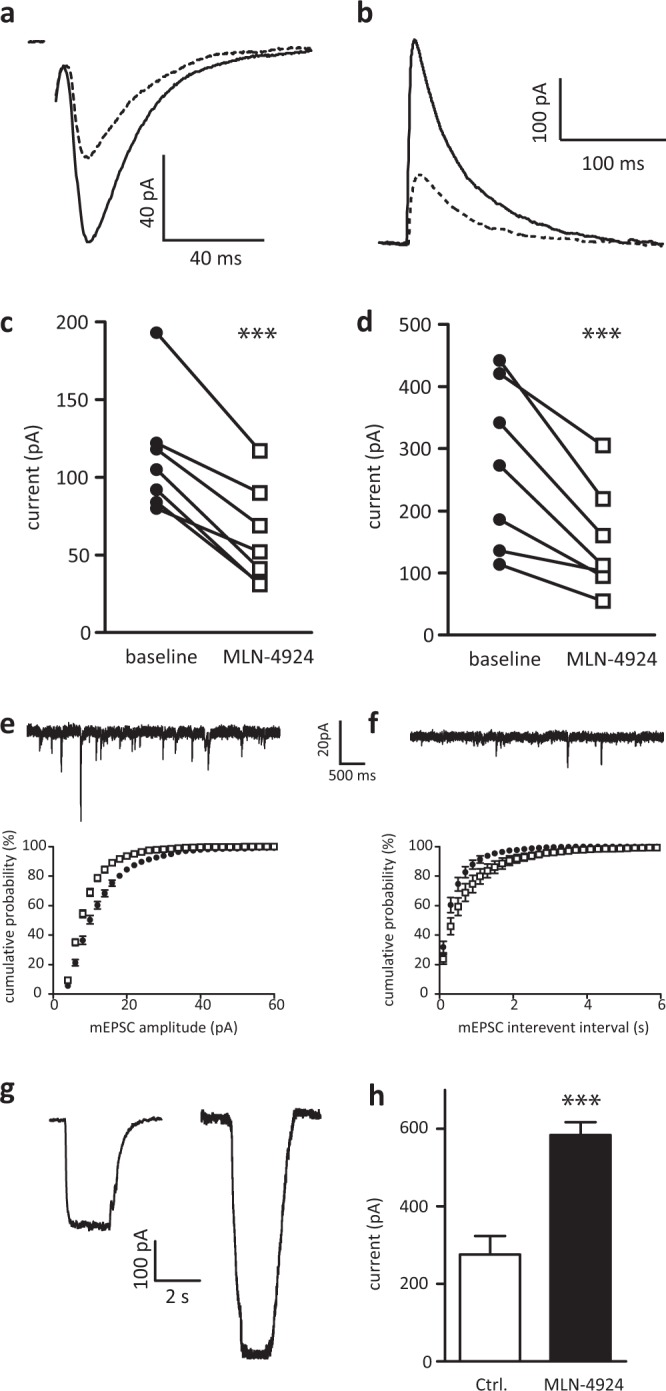
MLN-4924 treatment reduces post-synaptic AMPA and NMDA currents. (**a**) Sample traces of evoked AMPA currents of the same cell without (solid line) and with MLN-4924 (dashed line). (**b**) Sample traces of evoked NMDA currents of the same cell without (solid line) and with MLN-4924 (dashed line). Please note, stimulus artefacts have been omitted for clarity. (**c**) Recordings from several cells show that AMPA currents decrease after >30 min wash in of MLN-4924 in all cells recorded. (**d**) Recordings from several cells show that NMDA currents decreased after >30 min of MLN-4924 treatment in all cells recorded. (**e**) sample trace of mEPSC recorded from control cells. (**f**) sample trace of mEPSC recorded from cells incubated in MLN-4924 for 60 min. bottom panels show cumulative distributions of mEPSC amplitude (left) and mEPSC inter-event interval (right); ctrl; closed circles, MLN-4924 open squares. (**g**) Traces of AMPA-evoked currents from outside-out somatic patches in control and MLN-4924 treated cells. MLN-4924 treatment significantly increases extrasynaptic AMPA-R responses.

Two major effects could cause the reduction in neurotransmission: 1. a decrease in presynaptic vesicle fusion and consequently decreased neurotransmitter release or 2. a decrease in post-synaptic receptor density. To get a more detailed image of the changes in synaptic transmission upon neddylation inhibition, we recorded evoked EPSCs (eEPSCs) from hippocampal CA1 pyramidal cells, which allowed us to analysing the effect of neddylation inhibition on AMPA or NMDA receptor mediated currents separately. After obtaining a stable baseline of AMPA or NMDA currents, 1 µM MLN-4924 was added to the bath solution. Inhibiting neddylation for 60 min decreased both, AMPA and NMDA receptors currents to the same extend (Fig. 4c,d, relative AMPA current 47 ± 5%, p = 0.0002, paired t-test, n = 9; relative NMDA currents 45 ± 5%, p = 0.0031, paired t-test, n = 10). We exclude a direct inhibitory effect of MLN-4924 on the receptors, as changes of the evoked AMPA or NMDA currents did only occur after a longer application of MLN-4924; if MLN-4924 would directly block the receptors, the evoked currents should be immediately reduced. To further corroborate changes in synaptic transmission, we recorded miniature EPSCs (mEPSCs). These recordings showed that, mEPSC amplitude and frequency were significantly reduced after incubation in MLN-4924 (Fig. 4e,f, mean mEPSC amplitude: Ctrl. 15.1 ± 0.6 pA, n = 11; MLN, 10.9 ± 0.4 pA, n ± 10, p < 0.001; mean mEPSC frequency: Ctrl. 2.9 ± 0.5 Hz, n = 11; MLN, 1.6 ± 0.3 Hz, n = 10, p < 0.001, Kolmogorov-Smirnov test). A change in the size of these events is thought to reflect a postsynaptic change in the response to the neurotransmitter. In contrast, a change in frequency is thought to represent a change in neurotransmitter release^14^.

Previously, we showed that the synaptic scaffolding protein PSD-95 is neddylated. Further, we demonstrated that a non-neddylated variant of PSD-95 does not anchor AMPA receptors at the synapse^3^. As knock-down of PSD-95 by shRNA redistributed AMPA receptors from synapses to extrasynaptic membranes^15^, it is tempting to speculate that blocking neddylation of PSD-95 has a similar effect. To measure AMPA receptor density at extrasynaptic membranes, we performed somatic (non-synaptic) outside-out patches from cells treated with MLN-4924 and compared them to control conditions. MLN-4924 treatment resulted in increased AMPA receptor mediated currents (Fig. 4g,h, Ctrl. 276 ± 48 pA, n = 6; MLN 584 ± 33 pA, n = 5, t-test, p = 0.0007). Together, these data suggest that blocking neddylation by MLN-4924 decreases the number of AMPA and NMDA receptors at postsynaptic sites, which could be explained via the interaction of both receptors with PSD-95.

### Neddylation controls presynaptic vesicular release probability

**Figure 5:**
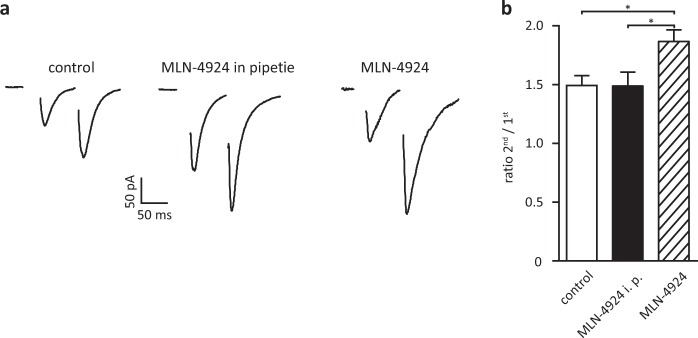
Release probability of presynaptic vesicles is decreased when neddylation is blocked. Release probability of excitatory synapses is decreased after MLN-4924 treatment. (**a**) Representative traces of typical EPSCs for control conditions (left), no effect when MLN-4924 is added only to the pipette solution (middle), increase in PPF when MLN-4924 is added to the bath (right). (**b**) Mean values of PPF in the different conditions. Please note, stimulus artefacts have been omitted for clarity.

Our previous biochemical data indicated that also presynaptic proteins are neddylated^3^. To test whether blocking neddylation affects presynaptic vesicular release, we recorded paired-pulse facilitation (PPF), a sensitive measure of changes in the probability of transmitter release, from CA1 neurons in acute hippocampal brain slices. PPF was increased in slices that were incubated in 1 µM MLN-4924 for 60 minutes. An increase in PPF correlates with a decrease in the probability of vesicular release from the presynapse. To limit the action of MLN-4924 only to the postsynapse, we added MLN-4924 to the pipette solution. This configuration did not affect PPF and proves that changes in PPF are specific for MLN-4924 action at the presynaptic side (Fig. 5a,b, PPF Ratio Ctrl. 1.52 ± 0.08, n = 10, MLN in pipette 1.49 ± 0.12, n = 9, MLN 1.87 ± 0.09, n = 9, Ctrl. vs. MLN p = 0.012, Ctrl. vs. MLN in pipette p = 0.83, t-test for each pair). These data demonstrate that blocking neddylation affects transmitter release.

### Neddylation is required to induce synaptic plasticity

So far, we demonstrated that basal synaptic transmission depends on neddylation. However, if neddylation of synaptic proteins is an important regulatory mechanism, activity dependent changes, e.g. synaptic plasticity, could be affected by neddylation. To test whether de-novo neddylation of synaptic proteins is required for synaptic plasticity, we applied MLN-4924 during the induction of LTP or LTD.

We recorded extracellular field potentials (fEPSPs) from stratum radiatum of the CA1 area and induced LTP by applying a brief tetanic stimulation of 100 Hz for 1 sec or induced LTD by stimulating with 1 Hz for 15 min. To block de-novo neddylation during the induction of synaptic plasticity, we added 1 µM MLN-4924 to the bath 5 min before the induction of plasticity until the end of induction. To monitor effects of MLN-4924 on synaptic transmission, we recorded from slices without inducing synaptic plasticity; here we never observed any changes in the slope of extracellular field potentials after these short times of neddylation inhibition.

**Figure 6:**
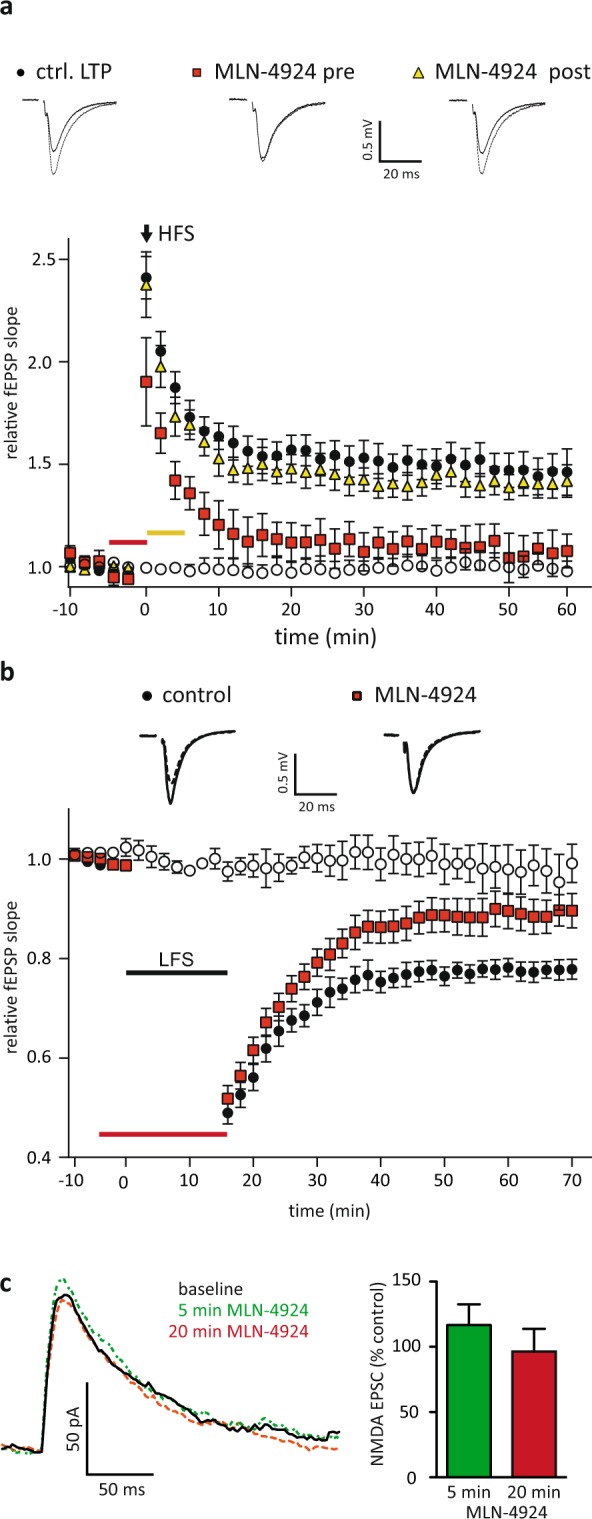
Neddylation is required for synaptic plasticity induction. (**a**) LTP was induced in acute hippocampal brain slices by high frequency stimulation (HFS). Application of 1 µM MLN-4924 5 min before starting induction (red bar) significantly decreased LTP compared to controls, while MLN-4924 added after the induction (yellow bar) did not affect LTP magnitude. (closed circles: control, red squares: MLN-4924 added before induction, yellow triangles: MLN-4924 after induction, open circles: MLN-4924 no LTP induction). (**b**) LTD could be induced under control conditions by low frequency stimulation (closed circles) and was strongly reduced when induced in the presence of MLN-4924 (red squares). Open circles show control fEPSP recordings from slices treated with MLN-4924 without inducing plasticity demonstrating that a short application of MLN-4924 does not directly affect synaptic transmission. Red bar indicates 1 µM MLN-4924 application. Insets on top show sample traces; solid line before induction, dashed line after induction. (closed circles: control, open squares: MLN-4924 before induction, open circles: MLN-4942 no LTD induction). (**c**) MLN-4924 does not directly inhibit NMDA receptor mediated currents. Please note, stimulus artefacts have been omitted for clarity.

When neddylation was inhibited 5 min before the induction of plasticity until the end of induction by 1 µM MLN-4924, the expression of LTP was significantly reduced. In contrast, blocking neddylation right after LTP induction for 5 min by 1 µM MLN-4924 did not affect LTP (Fig. 6a, Ctrl. LTP 1.46 ± 0.01, n = 10; MLN-4924 after induction 1.41 ± 0.01, n = 8; MLN-4924 during induction 1.06 ± 0.01, n = 10; no LTP induction 0.99 ± 0.01, n = 7; Ctrl. vs MLN-4924 after p = 0.21, Ctrl. vs MLN-4924 during p < 0.0001, Ctrl. vs no LTP p < 0.0001, ANOVA). The magnitude of LTD was strongly reduced when induced in the presence of MLN-4924 (Fig. 6b, Ctrl. 0.78 ± 0.02, n = 10; MLN during induction 0.89 ± 0.03 n = 10; no LTD induction 0.98 ± 0.03, n = 6; Ctrl. vs. MLN-4924 p < 0.0001, Ctrl. vs. no LTD p < 0.0001, ANOVA). To exclude that MLN-4924 directly blocks NMDA receptors within the time applied, which would prevent the induction of synaptic plasticity, we recorded evoked NMDA currents by whole cell patch recordings for CA1 pyramidal cells. NMDA currents were not inhibited by washing in 1 µM MLN-4924 (Fig. 6c normalized NMDA currents, 5 min MLN 111 ± 14%, p = 0.74, n = 7; 20 min MLN 91 ± 7%, p = 0.93, n = 5; ANOVA). Please note, the effects of MLN-4924 described in Fig. 4b were obtained after 60 min MLN-4924 application. These recordings clearly demonstrate that de-novo neddylation of target proteins during the induction phase of synaptic plasticity is required for the expression of hippocampal LTP and LTD.

## Discussion

Besides PSD-95, parkin, and PINK1 no other neddylated neuron specific proteins have been identified so far^3,12^. To get a better picture of neuronal functions affected by neddylation and thereby guiding the search for neddylated neuronal proteins, we characterized the effect of neddylation on neurotransmission. In contrast to dissociated neuronal cultures, spine size did not shrink within 120 min of MLN-4924 treatment in acute hippocampal slices.

Post-translational modifications of synaptic proteins are described to tightly regulate synaptic strength by modifying protein stability and function^16^. In a previous study, we reported that beyond classical post-translational modifications like phosphorylation or ubiquitylation, pre- and postsynaptic proteins are neddylated^3^. In that study, we found that neddylation of synaptic proteins is essential for spine development and spine stability in mature neurons. Similar results were obtained by the Patrick lab, showing that neddylation inhibition by pharmacological blocking of the activating enzyme NAE1 of the neddylation pathway by MLN-4924 affected spine morphology and synaptic strength^17^. Both studies used neuronal cultures to examine basal synaptic transmission and analysed neuronal transmission at time points when neddylation inhibition already affected spine morphology.

We here demonstrate that neddylation of synaptic proteins is essential for basal synaptic neurotransmission and synaptic plasticity. We have functional evidence that proteins on either side of the synapse are neddylated since blocking neddylation affects pre-synaptic release probability and post-synaptic receptor density. Our results indicate that neddylation of presynaptic proteins impacts on vesicular release probability. Therefore, proteins involved in vesicle fusion are good candidates^18^, which can be individually tested for neddylation by co-purification experiments. The situation at the postsynaptic side is slightly different, as PSD-95 was identified as the first neddylated synaptic protein in a previous study^3^. PSD-95 binds directly NMDA receptors and interacts via Stargazin with AMPA receptors. Changes in PSD-95 expression strongly regulate synaptic transmission, and overexpression of PSD-95 blocks LTP and increases LTD^19^. This central function of PSD-95 in postsynaptic function could directly explain the reduction of evoked AMPA EPSCs and the impaired LTP. Further, our results show that LTP and LTD were blocked by the application of MLN-4924 during the induction of synaptic plasticity. Importantly, applying MLN-4924 after the induction of LTP did not affect LTP magnitude, indicating that acute neddylation of synaptic proteins is required for the expression of activity dependent changes of synaptic transmission. Interestingly, blocking neddylation for up to 60 min did not affect neuronal excitability, which mainly depends on the proper function of voltage gated K^+^ and Na^+^ channels^20^. These data suggest that an effect of neddylation on voltage-gated ion channels is unlikely.

The reasons why no other neuronal proteins besides PSD-95, parkin and PINK1 have been identified as neddylated are manifold. The identification of neddylated proteins is challenging. Mainly, neddylation and de-neddylation are a rapid process, leading to a small amount of the neddylated form of a protein, which makes detection difficult. Unfortunately, a de-neddylase inhibitor has not been found so far; it would facilitate the search for neddylated proteins as substances like MG132 helped to identify ubiquitylated proteins. Next, the identification of neddylated lysines can be identified by mass spectrometry by detecting the mass shift of modified peptides caused by di-Gly overhangs after trypsin digestion; however, ubiquitin and Nedd8 generate the same di-Gly remnant, precluding discrimination between ubiquitin- and Nedd8-modified peptides^3,21,22^.

We sought here to get a more detailed understanding of what is functionally regulated by neddylation before spine morphology deteriorates. We showed evidence for pre- and postsynaptic regulation by neddylation and can exclude voltage gated ion channels that regulate membrane potential and excitability. The sensitivity of synaptic plasticity suggests that neddylation of synaptic proteins plays an important role in the activity dependent regulation of synaptic transmission.

Based on the findings presented here and based on the biochemical analysis of pre- and postsynaptic proteins presented before^3^, we conclude that several synaptic proteins are neddylated. Further, we show that de-novo neddylation is required for synaptic plasticity, as inhibiting neddylation before the induction of synaptic plasticity blocks the expression of synaptic plasticity while adding MLN-4924 after the induction of LTP has no effect on the magnitude of LTP.

So far, only little is known about neddylation beyond cullin-RING ligases. Leading to one obvious question: Could an indirect effect of reduced ubiquitylation caused by reduced neddylation of E3-ligases explain our observations? Cullin-RING ligases, the largest class of RING ubiquitin E3 ligases, are the best-characterized and fully validated neddylation substrates^23^. Hence, blocking neddylation could decrease the activity of these E3 ligases and in turn reduce ubiquitylation. If proteasomal degradation is the main regulator of synaptic function, blocking the ubiquitin-proteasome system (UPS) should have similar effects as blocking neddylation. We observed a decrease in both frequency and amplitude of mEPSC; however, it was shown that blocking the UPS by MG132 or inhibiting the ubiquitin E1 by ziram^24^ lead to an increase in mEPSC frequency and had no effect on mEPSC amplitude^25^. It was also discussed hat ubiquitylation has additional functions to protein degradation via the UPS and could regulate protein function or protein-protein interaction^25^, similar to the proposed function of neddylation^4^. In summary, our knowledge about the function of neddylation and ubiquitylation is still sparse. Therefore, identifying additional substrates, analysing changes in post-translational modifications, and their consequences on protein function will be necessary to further understand the complex regulation not only of synaptic function.

## Experimental Procedures

### Methods

#### Ethical statement

All animal experiments were performed in accordance with the relevant guidelines and regulations. Protocols were approved by the Landesamt für Natur, Umwelt und Verbraucherschutz Nordrhein-Westfalen.

#### Animals

C57BL/6 mice were group-housed under standard laboratory conditions with 12 hours light-dark cycle with food and water ad libitum. For tissue preparation, animals were handled in agreement with the European Union and local guidelines.

#### Hippocampal slice preparation for electrophysiological recordings and imaging

Acute hippocampal brain slices were prepared from P14-P19 C57BL/6 wildtype mice or expressing eGFP (GFP-M line^13^) for imaging. Animals were anesthetized with Isoflurane (Baxter), decapitated, brains were removed and placed into chilled and carbogen (95% O_2_, 5% CO_2_) gassed artificial cerebrospinal fluid (ACSF) containing in mM: 125 NaCl; 2.6 KCl; 1.4 MgSO_4_; 2.5 CaCl_2_; 1.1 NaH_2_PO_4_; 27.5 NaHCO_3_ and 11.1 D-glucose; pH 7.2, 310 mosm/kg. The hippocampus was transversally cut into 400 µm slices (VT1200S, Leica). Afterwards slices were equilibrated in ACSF for 30 min at 32 °C and thereafter kept at room temperature until recording or imaging.

#### Imaging

Imaging was performed using a custom built two-photon-microscope, operated with ScanImage^26^ and a Ti:sapphire Laser (Chameleon Vision-S, Coherent) tuned to 910 nm for GFP excitation. Slices were perfused with ACSF and image stacks of 2nd or 3rd order dendrites from pyramidal neurons were taken every 5 min with a 40x objective (LUMPlanFl, Olympus). After baseline imaging MLN-4924 treatment was started and images were obtained for 120 min. Spine sizes were analysed using custom-written software (MATLAB, Mathworks) and imageJ (NIH). Briefly, image stacks were maximum projected and for each spine the brightest pixels were averaged, divided by the average pixel values of their parent dendrite and normalized to the baseline images.

#### Electrophysiological recordings

Recordings were performed as described earlier^27^. Field excitatory postsynaptic potentials (fEPSP) were recorded from acute hippocampal brain slices. To avoid recurrent excitation, Schaffer collaterals were severed between CA3 and CA1. Synaptic responses were evoked by stimulating Schaffer collaterals at 0.33 Hz with 0.2 ms pulses and recorded in the stratum radiatum of CA1. fEPSP data were acquired using a Multiclamp 700B amplifier (Axon Instruments) and digitized with a Digidata 1440 A (Axon Instruments). All experiments were conducted at room temperature. The recording chamber was continuously perfused with carbogenated ACSF. Whole-cell patch recordings were obtained in ACSF supplemented with picrotoxin (100 µM) to block GABA_A_ receptors. Glass electrodes were filled with an internal solution containing (in mM): 150 Cs-gluconate, 8 NaCl, 2 MgATP, 10 HEPES, 0.2 EGTA, 0.1 spermine, and 5 QX-314, pH 7.2. NMDA currents in CA1 pyramidal cells were obtained by evoking eEPSC at + 40 mV, the current being taken 70 ms after stimulus. Series resistances were monitored during the experiment and typically ranged from 8 to 12 MΩ. Leak current was typically below 50 pA. Recordings were terminated if the series resistance exceeded 14 MΩ or the leak current exceeded 75 pA. Miniature EPSCs (mEPSCs) were recorded at −70 mV in ACSF supplemented with TTX (0.2 µM), PTX (100 µM), and trichlormethiazide (250 µM) to increase mEPSC frequency. Somatic outside-out patches were clamped at −70 mV. AMPA-R currents were evoked by local application of 500 mM S-AMPA for 2 sec in the presence of 100 mM trichlormethiazid. For current-clamp recordings pipette solutions contained the following (in mM): 150 K-methylsulphonate, 4 KCl, 4 NaCl, 4 MgATP, 0.4 MgGTP, and 10 HEPES. Current injections ranged from −100 to + 250 pA, increased in 25 pA steps. AP threshold in ΔmV was calculated as the difference between the resting membrane potential and the potential at which the first derivative of single APs became maximal.

#### Induction protocols for synaptic plasticity

After 10 min of stable baseline LTP was induced by tetanic stimulation (100 Hz for 1 s), LTD was induced by low frequency stimulation (1 Hz for 15 min). MLN-4924 treatment was started 5 min before synaptic plasticity induction and was stopped at the end of the induction protocol.

#### Western-Blot analysis

HEK cells treated for 60 minutes with either DMSO or MLN were briefly washed with PBS, harvested, lysed with hot non-reducing sample buffer and homogenized using microtip-aided sonication. SDS-polyacrylamide gel electrophoresis and immunoblotting were performed using standard procedures. The following antibodies were used: Rabbit anti-UBE2M 1:1,000 (clone EPR5333, abcam), mouse anti-UBE2C 1:1,000 (clone 1F5D3, abcam) and fluorescence-linked secondary antibodies 1:2,000 (Li-cor). Fluorescence signals were detected using an Odissey Fc System (Li-cor, Lincoln, Nebraska USA).

#### Statistical analysis

For each experiment, including recordings and imaging experiments, data were collected from at least three independent animal preparations. Data were tested for normal distribution and statistical significance using Prism 5 (GraphPad, San Diego, CA). Error bars represent SEM. We used paired student’s t-tests to compare paired data and unpaired t-tests for comparison the means of different groups. We used ANOVA to compare multiple groups. Significance levels are denoted as follows: *p < 0.05; **p < 0.01; ***p < 0.001.

## Supplementary information

Figure S1

## Data Availability

Data generated in this study are available from the corresponding author upon request.
