# Supplementary material for: Neddylation regulates excitatory synaptic transmission and plasticity
Source: Sci Rep. 2019 Nov 29;9:17935. doi: 10.1038/s41598-019-54182-2 (PMC6884593; doi:10.1038/s41598-019-54182-2)
Supplement: Supplementary file 1 — Figure S1 [file 41598_2019_54182_MOESM1_ESM.pdf]

Neddylation regulates excitatory synaptic transmission and plasticity

Marisa M. Brockmann, Michael Döngi, Ulf Einsfelder, Nils Körber, Damian Refojo, and Valentin Stein

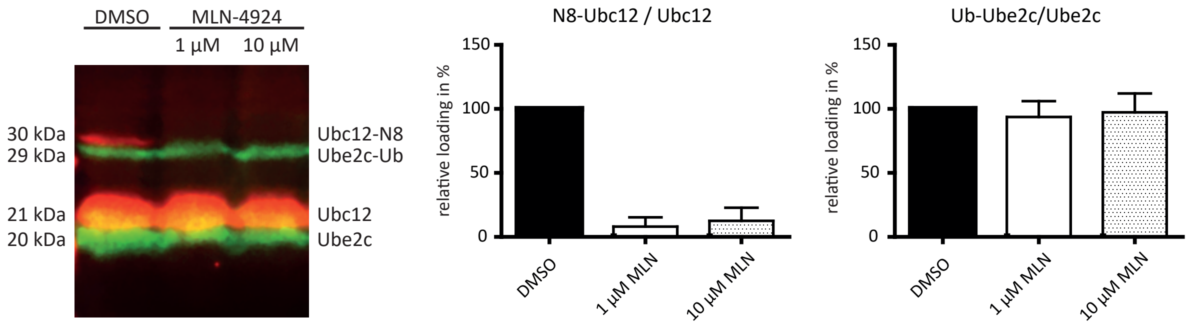

HEK293T cells treated for 60 min with DMSO (vehicle), 1 µM MLN-4924 or 10 µM MLN-4924. Whole cell lysates were subjected to Western Blot and stained against Ube2c (green) and Ubc12 (red) at the same time. Bands around the size of 20 kDa show non-loaded enzyme, bands around 30 kDa show enzyme loaded with Ubiquitin (Ub) or Nedd8 (N8). Application of MLN-4924 reduces the amount of loaded Ubc12 below the detection threshold, while the amount of loaded Ube2c is unaffected, even when using a higher concentration of 10 µM MLN-4924 (Ubc12: DMSO 100%; 1 µM MLN 8.1 ± 7.2 %, $p = 0.0002$, $N = 5$; 10 µM MLN 12.6 ± 10.3 %, $p = 0.001$, $N = 5$; Ube2c: DMSO 100%; 1 µM MLN 93.7 ± 12.4 %, $p = 0.63$, $N = 5$; 10 µM MLN 97.3 ± 14.8 %, $p = 0.86$, $N = 5$).

Brockmann et al.
Supplementray Figure S1
